# Relationship between cognitive impairment and oxidative stress markers in patients with early-onset schizophrenia

**DOI:** 10.12669/pjms.41.3.9999

**Published:** 2025-03

**Authors:** Rongli An, Xianli Zhang, Xianglei Luo, Zhongxia Zhao, Xiaohong Zhen

**Affiliations:** 1 Rongli An, Department of Psychiatry, The Eighth Hospital of Shijiazhuang, Shijiangzhuang 050080, Hebei, P.R. China; 2 Xianli Zhang, Department of Pharmacy, The Eighth Hospital of Shijiazhuang, Shijiangzhuang 050080, Hebei, P.R. China; 3 Xianglei Luo, Department of Psychiatry, Bazhou Traditional Chinese Medicine Hospital, Bazhou 050080, Hebei, P.R. China; 4 Zhongxia Zhao, Department of Psychiatry, The Eighth Hospital of Shijiazhuang, Shijiangzhuang 050080, Hebei, P.R. China; 5 Xiaohong Zhen, Department of Psychiatry, The Eighth Hospital of Shijiazhuang, Shijiangzhuang 050080, Hebei, P.R. China

**Keywords:** Early-onset, Schizophrenia, Cognitive impairment, Oxidative stress marker

## Abstract

**Objective:**

To investigate the relationship between cognitive impairment and oxidative stress markers in patients with early-onset schizophrenia(EOS).

**Methods:**

This was a retrospective study. A total of 42 patients with EOS admitted to The Eighth Hospital of Shijiazhuang from December 2021 to December 2023 and 42 healthy volunteers were included in this study and classified as the observation group and the control group. Cognitive impairment and oxidative stress markers were assessed in both groups. The Measurement and Treatment Research to Improve Cognition in Schizophrenia(MATRICS) Consensus Cognitive Battery(MCCB) was used to evaluate cognitive function, and the serum levels of superoxide dismutase(SOD), malondialdehyde, glutathione peroxidase(GSH-Px), catalase, homocysteine, and nitric oxide(NO) were measured. Pearson correlation analysis was conducted to clarity the relationship between cognitive function and oxidative stress markers.

**Results:**

The SOD and GSH-Px levels in the observation group were lower than in the control group, while homocysteine, catalase, malondialdehyde, and NO levels were higher in the observation group. Compared to the control group, patients in the observation group scored significantly lower for information processing speed, attention/vigilance, working memory, verbal learning, visual learning, reasoning and problem solving, and social cognition (*P*< 0.01, respectively). SOD and malondialdehyde levels were found to have a significant negative correlation with information processing speed, with r values of -0.342 and -0.350, respectively (*P* < 0.05).

**Conclusion:**

Cognitive impairment in patients with EOS appears to be associated with oxidative stress. There is a significant negative correlation between the levels of SOD and malondialdehyde and information processing speed.

## INTRODUCTION

Schizophrenia is a severe chronic mental illness characterized by cognitive impairment, behavioral disturbances, hallucinations, delusions, affective disorders, and thought disorders. It ranks among the top 15 leading causes of disability worldwide.^1^ Individuals with schizophrenia have an average life expectancy approximately 15 years shorter than the general population, with a 5% to 10% risk of suicide over their lifetime.^2^ In China, the estimated lifetime prevalence of schizophrenia is 0.54%, with a disability prevalence of 0.41%.^3^

Schizophrenia is associated with cognitive impairment, affecting working memory, attention, visual memory, and language learning ability.^4^ Approximately 98% of individuals with schizophrenia experience cognitive impairment, significantly impacting overall functioning and impeding rehabilitation.^5^ Schizophrenia manifests at different ages with notable variations in symptoms.^6^ Early-onset schizophrenia (EOS) refers to patients who exhibit symptoms of schizophrenia before the age of 18, constituting approximately 11% of schizophrenia cases.^7^ Patients with EOS experience incomplete brain development and more severe cognitive impairments, leading to a poor prognosis.^8^

The etiology of schizophrenia remains unclear, with evidence suggesting a strong interplay between genetic and environmental insults, such as discrimination, financial difficulties, adverse childhood events, adolescent substance abuse, maternal stress, infections, pregnancy complications, and nutritional deficiencies.^9^ These various detrimental factors for individuals with schizophrenia are broadly categorized into several biopathophysiological hypotheses, with the most significant being neurotransmitter imbalances, oxidative/reductive damage, neuroinflammation, and mitochondrial dysfunction.^10^ This study investigated the relationship between cognitive impairment and oxidative stress markers in patients with EOS.

## METHODS

This was a retrospective study. A total of 42 patients with EOS admitted to The Eighth Hospital of Shijiazhuang from December 2021 to December 2023 and 42 healthy volunteers were included in this study and classified as the observation group and the control group. The sample size required for each group was calculated by the formula.


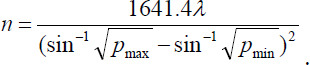


### Ethical Approval:

The study was approved by the Institutional Ethics Committee of the Eighth Hospital of Shijiazhuang (No.2022-9-18: Dated: December 1st, 2023), and written informed consent was obtained from all participants.

### Inclusion criteria:

- Patient age ≤ 18 years.
- Initial diagnosis of EOS, with a duration of more than six months but within two years.
- Provision of signed informed consent forms by legal guardians, and voluntary participation in the study.
- Patients with a Positive and Negative Syndrome Scale(PANSS) score ≥ 60.
- Ability to communicate and cooperate with the research.
- No prior treatment with psychiatric drugs or interventions such as electroconvulsive therapy.
- No history of smoking, alcohol consumption, or drug use.

### Exclusion criteria:

- Presence of other psychiatric or cognitive disorders.
- Patients with severe illnesses such as malignant tumors or cardiovascular diseases.
- Participants who withdrew from the study prematurely.

### Control group:

In addition to the inclusion criterion of no psychiatric illness as confirmed by professionals, the inclusion and exclusion criteria were the same as those specified in observation group. The observation group comprised 21 males and 21 females, with an average age of (15.90±1.45) years, ranging from 13 to 18 years. The control group also consisted of 21 males and 21 females, with an average age of (16.21±1.49) years, ranging from 13 to 18 years. There were no statistically significant differences in the general data between the two groups (all *P* > 0.05).

### Comparison of cognitive function:

Cognitive function was assessed using the Measurement and Treatment Research to Improve Cognition in Schizophrenia (MATRICS) Consensus Cognitive Battery (MCCB).^11^ Cognitive function was measured across seven cognitive domains: information processing speed, attention/vigilance, working memory, verbal learning, visual learning, reasoning and problem solving, and social cognition. Trained psychiatrists were assigned to evaluate the performance of patients with EOS in the MCCB tests and recorded the results.

### Comparison of oxidative stress marker levels:

Both groups underwent fasting venous blood collection at 8 am, with a blood sample volume of five mL. After mixing with the anticoagulant ethylenediamine tetraacetic acid (EDTA), the samples were centrifuged at 3000 rpm, 4°C, for 20 minutes. After centrifugation, serum was separated and stored at -80°C for later use. Enzyme-linked immunosorbent assay (ELISA) was conducted to determine the activity levels of serum superoxide dismutase (SOD), malondialdehyde, glutathione peroxidase (GSH-Px), catalase, homocysteine, and nitric oxide (NO).

### Statistical analysis:

Statistical analysis was performed using the software SPSS 22.0. Measurement data were presented as “mean ± standard deviation (*χ̅*±*S*)”. The independent sample t-test was employed for comparisons, and *p*< 0.05 indicated statistically significant differences. The relationship between cognitive function and oxidative stress markers was assessed via Pearson correlation analysis.

## RESULTS

As shown in Table-I, patients in the observation group score significantly lower for information processing speed, attention/vigilance, working memory, verbal learning, visual learning, reasoning and problem solving, and social cognition compared to the control group (*P*< 0.05, respectively). Comparison of oxidative stress marker levels. As shown in Table-II, compared to the control group, patients in the observation group exhibit significantly lower levels of SOD and GSH-Px and significantly higher levels of catalase, homocysteine, malondialdehyde, and NO (*P* < 0.05, respectively). As shown in Table-III, most of the oxidative stress markers in patients with EOS display negative correlations with cognitive function. Notably, SOD and malondialdehyde levels exhibit a significant negative correlation with information processing speed (*P* < 0.05, respectively).

**Table-I:** Comparison of cognitive function scores (n = 42,*χ̅*±*S*).

Group	Control group	Observation group	t	P
Information processing speed	33.90±2.60	31.76±1.92	4.292	5.192E-5
Attention/vigilance	36.64±2.97	29.50±5.32	7.602	1.575E-10
Working memory	38.81±6.36	29.02±4.12	8.371	3.708E-12
Verbal learning	44.31±4.13	39.19±2.77	6.666	4.624E-9
Visual learning	31.93±1.77	30.29±0.86	5.400	1.226E-6
Reasoning and problem solving	39.71±2.44	32.38±2.27	14.243	7.771E-24
Social cognition	37.38±4.04	33.74±3.64	4.342	4.005E-5

**Table-II:** Comparison of oxidative stress marker levels (n = 42, *χ̅*±*S*).

Group	SOD (U/mL)	GSH-Px (U/mL)	Catalase (U/mL)	Homocysteine (μmol/L)	Malondialdehyde (nmol/L)	NO (μmol/L)
Control group	65.97±1.40	90.54±16.31	6.35±2.08	12.03±2.58	3.08±0.24	34.77±8.16
Observation group	61.17±3.55	62.47±9.61	10.05±1.67	20.79±4.07	5.33±1.14	56.18±5.61
*t*	8.143	9.610	-8.993	-11.763	-12.539	-14.012
*P*	6.234E-11	3.431E-14	1.078E-13	3.481E-18	3.031E-16	2.422E-22

**Table-III:** Relationship between cognitive function scores and oxidative stress markers (n = 42).

Marker	SOD	GSH-Px	Catalase	Homocysteine	Malondialdehyde	NO
Information processing speed	-0.342*	-0.037	-0.072	-0.123	-0.350*	-0.047
Attention/vigilance	-0.044	-0.261	0.061	0.046	-0.136	-0.084
Working memory	-0.035	-0.035	-0.282	-0.221	0.034	0.024
Verbal learning	-0.086	-0.139	-0.093	-0.302	0.067	-0.116
Visual learning	-0.088	-0.114	0.107	0.051	0.009	-0.154
Reasoning and problem solving	-0.013	-0.115	0.085	-0.138	0.087	-0.048
Social cognition	-0.017	-0.081	-0.232	-0.083	0.127	-0.218

* P < 0.05

## DISCUSSION

Schizophrenia is a chronic brain disorder affecting approximately 1% of the global population, imposing significant health and financial burdens on society.^12^ Traditionally, schizophrenia has been hypothesized as a disease characterized, in part, by abnormal changes in brain structure and connectivity. People with EOS usually develop in young adulthood and are affected by environmental factors, drugs and unhealthy habits for a shorter period of time than those with late-onset schizophrenia. And the cognitive ability of such patients is relatively less affected by the disease itself. This makes patients with EOS an ideal population for investigating the relationship between oxidative stress levels and cognitive function. In order to mitigate the influence of confounding factors, this study specifically selected patients who experienced their first onset for more than six months but had a disease course of less than two years. Additionally, these patients had not received relevant medication or treatment like electroconvulsive therapy. The inclusion criteria also encompassed the absence of a history of smoking, alcohol consumption, or drug use to eliminate the potential impact of treatment, long-term disease progression, and unhealthy habits on the cognitive function of the patients.

Several studies have indicated a potential association between the dysregulation of the oxidative stress system and cognitive impairment in patients with schizophrenia.^13^ Oxidative stress is defined as a sustained imbalance between toxic reactions involving oxygen and nitrogen and the body’s antioxidant defenses, which is essential for normal physiological functioning.^14^ However, abnormal levels of oxidative stress markers can damage biomolecules and result in various diseases. Within cell membranes, hydroxyl radicals can initiate lipid peroxidation and induce changes in cell membrane structure and function. Given the lipid-rich brain structure, high oxygen consumption, and a lack of sufficient antioxidant barriers, the brain is particularly prone to oxidative imbalance.^15^

Under physiological conditions, cells are primarily protected by the coordinated and sequential interactions of SOD, GSH-Px, catalase, homocysteine, malondialdehyde, and NO from oxidative damage. These enzymes are crucial components of the antioxidant defense system (AODS).^16^ Antioxidant enzymes like SOD, catalase, and GSH-PX play a vital role in clearing free radicals and preventing oxidative damage to brain cells.^17^ homocysteine can inhibit the expression of proteins such as Bcl-2 and promote the expression of proteins like Caspase and Bax, leading to pronounced necrosis and apoptosis of nerve cells. These changes alter the network structure of the nervous system and disrupt normal neural development.^18^ Excessive NO can cause cell damage, resulting in impaired neural circuits and cognitive impairment in patients.

According to the experimental results, the observation group exhibited lower levels of SOD and GSH-Px compared to the control group, while homocysteine, catalase, malondialdehyde, and NO levels were higher in the observation group. Micó JA et al. found that the decreased antioxidant defense ability in patients with early-onset first psychotic episode can be measured by reduced Total antioxidant status and glutathione levels. Lipid damage and glutathione peroxidase activity were higher in patients than in controls.^19^ These results support the conclusions of this study. Additionally, compared to the control group, the observation group scored lower for information processing speed, attention/vigilance, working memory, verbal learning, visual learning, reasoning and problem solving, and social cognition. Therefore, the occurrence of EOS appears to be associated with changes in oxidative stress levels. The results of the study by Li J et al. suggest that improving oxidative stress status in patients with schizophrenia may enhance their cognitive function, which also supports the conclusions of this study.^20^

The relationship between cognitive function and oxidative stress markers was investigated using Pearson correlation analysis. A significant negative correlation was observed between SOD and information processing speed. SOD plays a crucial role in converting superoxide radicals into O_2_ and H_2_O_2_, with H_2_O_2_ subsequently being transformed into H_2_O and O_2_ through the action of catalase and GPX. Disruption in the oxidative stress system, characterized by a decrease in SOD levels and a reduction in endogenous antioxidants, can lead to DNA damage or alterations in the structure of brain cell membranes. This, in turn, results in damage to neural circuits, affecting the cognitive function of patients with schizophrenia. catalase activity in patients with schizophrenia is relatively weak, contributing to elevated oxidative stress levels and thus participating in disease progression.^21^

A significant negative correlation was observed between malondialdehyde and information processing speed. malondialdehyde is an active aldehyde produced after lipid peroxidation.^22^ An malondialdehyde level exceeding the normal range indicates enhanced efficiency of oxygen free radical reactions and signifies an increase in oxygen free radicals as one of the reactants. Therefore, changes in malondialdehyde levels indirectly reflect alterations in oxygen free radical content. Elevated levels of free radicals and malondialdehyde beyond normal levels in the body or within cells lead to reduced mitochondrial function, enhancing excitotoxicity, promoting inflammation, and exacerbating peroxidative damage to neuronal DNA, lipids, and proteins. This process represents one of the fundamental mechanisms of brain injury, consequently influencing the information processing speed of the brain. This study provides valuable data supporting the understanding of the relationship between cognitive impairment and oxidative stress markers in patients with schizophrenia, offering insights for early diagnosis and intervention.

### Limitations:

However, patients with schizophrenia often experience prolonged illness duration before seeking treatment and may have undergone certain drug interventions. All this can affect oxidative stress levels. Therefore, further investigations are warranted to explore how confounding factors may affect oxidative stress levels. Understanding the impact of such factors is crucial to enhancing the accuracy of using oxidative stress levels as a diagnostic tool for schizophrenia.

## CONCLUSIONS

In conclusion, cognitive impairment in patients with EOS appears to be associated with oxidative stress. There was a significant negative correlation between the levels of SOD and malondialdehyde and information processing speed.

### Authors’ Contributions:

**RA** and **XZ:** Carried out the studies, participated in collecting data, and drafted the manuscript, and are responsible and accountable for the accuracy or integrity of the work.

**XL, XZ** and **ZZ:** Literature search, performed the statistical analysis and participated in its design. Critical Review.

All authors read and approved the final manuscript.
